# XRD-Thermal Combined Analyses: An Approach to Evaluate the Potential of Phytoremediation, Phytomining, and Biochar Production

**DOI:** 10.3390/ijerph16111976

**Published:** 2019-06-04

**Authors:** Dario Fancello, Jessica Scalco, Daniela Medas, Elisa Rodeghero, Annalisa Martucci, Carlo Meneghini, Giovanni De Giudici

**Affiliations:** 1 Department of Chemical and Geological Sciences, University of Cagliari, Cittadella Universitaria, 09042 Monserrato, Italy; darfan@hotmail.it (D.F.); jescalco91@gmail.com (J.S.); gbgiudic@unica.it (G.D.G.); 2 Department of Physics and Earth Sciences, University of Ferrara, I-44100 Ferrara, Italy; elisa.rodeghero@unife.it (E.R.); annalisa.martucci@unife.it (A.M.); 3 Department of Sciences, University Roma Tre, 00146 Rome, Italy; carlo.meneghini@uniroma3.it

**Keywords:** *Juncus acutus*, metal pollution, phytoremediation, conventional XRD, synchrotron-based XRD, thermal analyses

## Abstract

A method for evaluating the potential of reuse of biomasses for economic purposes is here presented starting from a case study. *Juncus acutus* plants and rhizospheres were harvested from abandoned Zn–Pb mine areas of southwest Sardinia (Italy). Thermogravimetry and Differential Thermal analyses were performed to evaluate the temperatures at which significant reactions occur. X-ray Diffraction (XRD) analysis was carried out on raw samples and on samples heated ex-situ (by a conventional diffractometer) or in-situ (by synchrotron-based diffraction). Raw samples mainly consist of quartz, phyllosilicates, and feldspars with minor amounts of sulfides, sulfates, and Fe, Pb, and Zn carbonates, concentrated in the rhizosphere. After heating, Zn and Fe oxides and willemite are observed in internal roots and stems, revealing the presence of these metals in the plant tissues. In-situ heating was less effective than ex-situ in revealing minor phases in organic samples, probably because the scarcity of oxygen within the sample holder did not allow the degradation of organic compounds and the oxidation of sulfides, resulting in a low quality XRD signal even if obtained with the high resolution ensured by a synchrotron light source. This method can be applied to plants from polluted sites for metal exploitation, and/or to biomasses from unpolluted sites for biochar production, since both applications take advantage of the knowledge of the minerals formed after heating.

## 1. Introduction

The chemical and biological interaction between roots and soil in contaminated environments is a complex research field that involves biology, geology, chemistry, and environmental engineering. Its significance comes from the multiple potential applications, from the determination of toxicity threshold for plants [1], to the transfer of contaminants in the food chain [2], to the use of plants in phytoremediation. The latter field has acquired increasing interest during the last thirty years and it is widely recognized as one of the most promising techniques for limiting the diffusion of pollutants [3,4,5,6,7]. Plants can act as effective removers for both organic and inorganic compounds through different processes as rhizofiltration [8], phytostabilization [9], phytoextraction [10], phytovolatilization [11], and phytotransformation [12].

Phytoremediation is especially suitable for trace metals contamination because, unlike organic compounds, metals cannot be decomposed and thus they should be removed or at least immobilized. Metal removal and immobilization by plants depend on many factors as the species of plants, the type and the extent of contamination, the hydrological regime, the geochemical features of soil, and so on [13,14]. A wide variety of parameters strongly interacting with each other must be carefully examined prior to attempting a successful phytoremediation intervention. Several papers explore the different sides of this complex topic: from the choice of the better species to remove the metals of interest [15], to the adaptation of the species to the polluted environment [16]; from the role of root exudates in the chemical behavior of metals in the rhizosphere [17], to the symbiosis between roots and mycorrhizae [18] and/or bacteria [19]; from the migration of metals within the plant [20,21], to their physiological role/effect in the plant growth [22], to the possibility of recover these metals for commercial purposes [23].

Several species, named as hyperaccumulator, are able to concentrate metals in their organic tissues without suffering phytotoxic effects [24], and most of them are selective for one or more metals [5] (and references therein). Great interest is focused on such plants since they can make the phytoremediation intervention economically viable through the recovery of valuable elements. Other species cannot accumulate significant amounts of metals in the aerial parts but can stabilize them in the rhizosphere or on the root epidermis, as demonstrated by previous investigations for *Vallisneria americana* [25], *Phragmites australis* [26], *Solanum lycopersicum* [27], *Lycopersicon esculentum* [28], *Euphorbia pithyusa* L. [29], *Pistacia lentiscus* [30], and *Juncus acutus* [20,31]. These species cannot be harvested for metal recovery but could be reused for the biochar production that in turn can be employed in soil amendment/remediation [32,33]. This application has different ecological outcomes such as, stabilizing pollutants, making productive unused polluted lands, and solving the problem of biomass storage that, even if weakly contaminated, could produce metal leaching.

Despite the significant content of metals in both hyperaccumulator and “metal tolerant” plants, it is quite difficult to detect trace metal phases within their tissues by common X-ray Diffraction (XRD) at room temperature because metals are mainly bound to organic ligands as proteins, amino acids, and organic acids [34,35,36] resulting in low or absent signals in XRD patterns. For this reason, heating organic tissues up to their combustion or pyrolysis could help to reduce the background produced by organic compounds allowing for detection of possible trace metal-bearing mineral phases already present in the plants, or it could favor the formation of new minerals. Furthermore, the progressive heating of samples combined with thermogravimetry (TG) and Differential Thermal Analyses (DTA) could allow for recognition of the optimum temperature at which a given phase forms. Different applicative fields require this kind of information, for instance, phytomining [37,38] in polluted environments and biochars production [32,33,39] in unpolluted ones.

*Juncus acutus* is a halophyte plant and is considered a pioneer species as it can grow in extreme environments as salt marshes, dunes, and metal-polluted areas [20,26,31]. It is well known for its tolerance to high concentrations of metals [40] and for its capability to stabilize them in the rhizosphere or in the external roots. In addition, it has been successfully tested in hydroponic culture for the removal of Zn, Cd, Ni, Cr, and organic compounds as bisphenol A and antibiotics [41]. The importance of this species is not given just by its performance in phytoremediation but also by its widespread distribution throughout almost all of Europe, the Mediterranean Basin, northern Africa, Caucasus, western Asia, and also Baja California and Australia. Thus, its adaptability to different extreme environments and its worldwide diffusion, makes *Juncus acutus* a suitable candidate to perform local experiments whose results can be applied elsewhere.

In this paper, we present a study performed on samples of *Juncus acutus* harvested in an abandoned Zn–Pb mine area from southwest Sardinia, (Italy). XRD analysis was performed on *Juncus acutus* samples at room temperature and after ex-situ and in-situ heating, combined with thermal analyses (TG and DTA). Four different aliquots were selected for each sample: rhizosphere, external root, inner root, and stem in order to investigate the mineralogy of the plant/soil system and how it is affected by heating processes.

## 2. Field Occurrence

The Montevecchio-Ingurtosu mine district (southwestern Sardinia, Italy) is a complex of mining sites exploited since the first half of the 1800s to the end of the 1900s, mainly for Pb and Zn extraction. The cessation of mining activities left behind millions of m^3^ of dumps, open pits, and a strong widespread metal pollution [42]. The mineralizations are hosted within the Variscan metamorphic basement and close to a carboniferous batholith known as Arburese granite [43] (and references therein). The primary mineralization, linked to the hydrothermal activity subsequent to the granite emplacement at shallow crustal levels [44], mainly consists of galena (PbS) and sphalerite (ZnS), with minor amounts of pyrite (FeS_2_) and chalcopyrite (CuFeS_2_) and accessory sulfide phases as arsenopyrite (FeAsS), greenockite (CdS), enargite (Cu_3_AsS_4_), argentite (Ag_2_S), etc. The oxidation of these minerals produced sulfates, carbonates, and hydroxides as smithsonite (ZnCO_3_), cerussite (PbCO_3_), malachite (Cu_2_(CO_3_)(OH)_2_), azurite (Cu_3_(CO_3_)_2_(OH)_2_), anglesite (PbSO_4_), hemimorphite (Zn_4_(Si_2_O_7_)(OH)_2_·H_2_O), goethite (FeO(OH)), etc. Lead mineralization is commonly associated with a quartz + barite gangue, whereas Zn mineralization is found within a carbonate gangue made up of calcite, dolomite, and ankerite.

**Figure 1:**
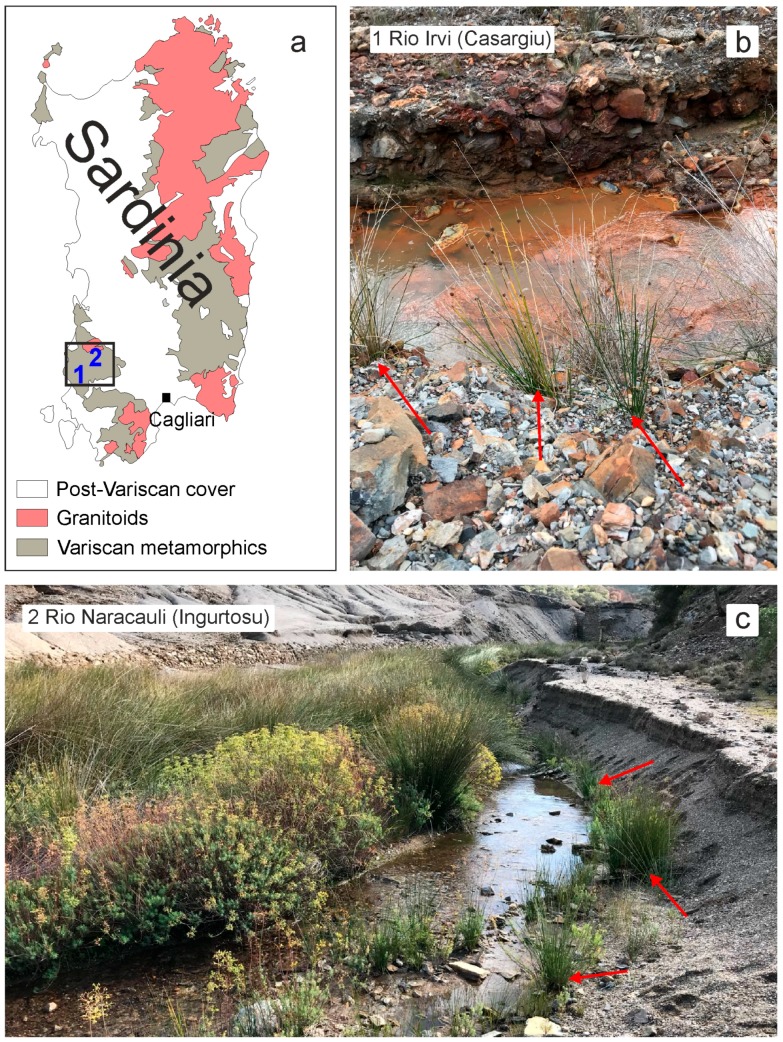
Schematic geological map of Sardinia (**a**). Sampling sites along Rio Irvi (**b**) and Rio Naracauli (**c**). Red arrows indicate the harvested plants.

The samples of *Juncus acutus* were harvested along the banks of two streams that flow within the above described mine district (Figure 1). The seasonal behavior of the two selected streams—Rio Irvi, Casargiu [45,46] and Rio Naracauli [47], Ingurtosu—is due to the climate of southern Sardinia, characterized by a long dry season starting in May and ending in September or October with minimum rainfalls in July and maximum in November [48]. Sampling was performed at the end of the dry period and thus with low levels in the streams.

## 3. Materials and Methods

**Figure 2:**
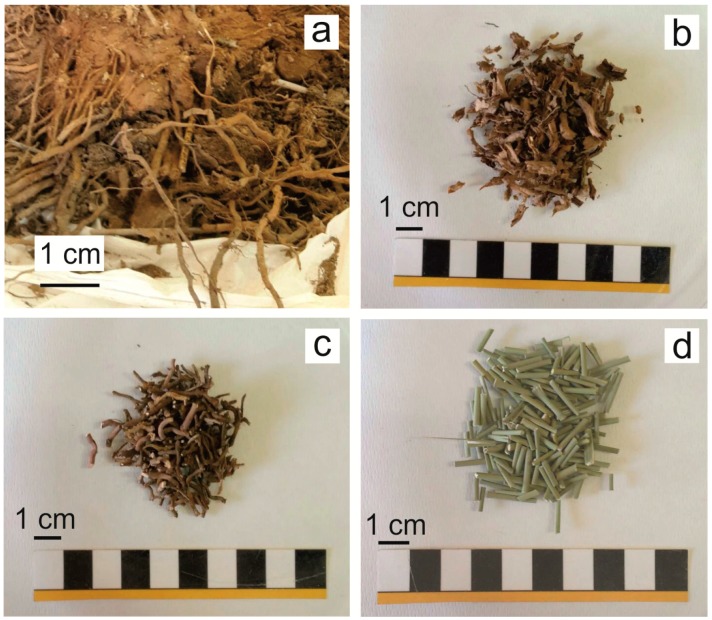
Images of rhizosphere (**a**), external surface of roots (epidermis) (**b**), internal roots (**c**), and stems (**d**) of *Juncus acutus* collected along the Rio Irvi (SW Sardinia).

Three samples of *Juncus acutus* including the whole plant, its roots and rhizosphere were collected from each sampling site. From each sample rhizosphere, external surface of roots (epidermis), internal roots (defined in this study as the roots without the epidermis) and stems (Figure 2) were manually separated with a scalpel under a reflected light microscope. Rhizosphere samples (here considered as the 2 mm of soil surrounding the roots) were dried at room temperature and grinded in an agate mortar whereas organic samples were washed with de-ionized water, dried at 40 °C and grinded in a mill (Retsch ZM200).

Clayey and silty fractions of rhizosphere samples were sequentially separated by the wet gravity separation method according to the “Stokes law”. De-ionized water was used as dispersive phase and sodium hexametaphosphate and sodium carbonate as deflocculant. This treatment was required to better characterize clayey minerals during XRD analyses.

The concentrations of Ca, Mg, Na, and K for all samples were determined through Inductively Coupled Plasma–Optical Emission Spectrometry (ICP-OES) analysis after acid digestion at the Department of Chemical and Geological Sciences of Cagliari University (Italy), according to the procedure reported by Medas et al. [20]. To evaluate the analytical precision of the acid digestion procedure, samples were digested and analyzed in duplicate. The accuracy was evaluated processing samples together with blanks and a reference material (polish Virginia Tobacco leaves—INCT-PVTL-6), prepared with the same mixture. For ICP-OES analysis, procedural blanks, standard solutions, and reference solutions (SRM 1643e and EnviroMAT Drinking Water, High EP-H-3 and Low EP-L-3) were analyzed after every five samples to estimate potential contaminations, accuracy, and precision. Also, minor elements’ data reported by Medas et al. [20] are considered.

Thermogravimetric and Differential Thermal Analyses were performed, at the Department of Physical and Earth Sciences of Ferrara University (Italy), on stem and rhizosphere samples from the Rio Irvi site in order to identify the main physical/mineralogical reactions occurring during the progressive heating as well as to understand their thermodynamic. Thermogravimetric (TG/DTG) and Differential Thermal Analysis (DTA) were simultaneously performed by Netzsch STA 409 instrument, with a constant 10 °C min−1 heating rate from 30 to 900 °C in streaming dry air.

Three kind of data were obtained: (i) TG gives quantitative information on the change in sample mass as a function of the temperature; (ii) DTA gives qualitative information on the endo- or exothermicity of transformations occurring during the thermal treatment; and (iii) the derivative of the TG curve (DTG) allows us to detect changes in the slope of the TG curve, occurring, for example when thermal events overlap, which might otherwise not be detected.

X-ray diffraction (XRD) analyses on both raw and heated samples harvested from Rio Irvi and Rio Naracauli, were performed following two different procedures. Samples from Rio Irvi were analyzed, at the Department of Physical and Earth Sciences of Ferrara University (Italy), before and after ex-situ heating at 120°, 300°, and 600 °C in a muffle under atmospheric air flux. Heating rate was about 10°C/min and the step temperature was kept for 30 minutes. XRD patterns were collected by a Bruker-AXS D8 diffractometer with Cu Kα radiation (λ = 1.54056 Å), equipped with the Soil-X detector; operating conditions were 40kV and 40 mA, 2θ range 5–80° and 0.02° of step size.

Samples from Rio Naracauli were analyzed before and during in-situ heating at the MCX (Materials Characterisation by X-ray diffraction) beamline (experiment number 20140061) of Elettra Synchrotron (Trieste, Italy) [49] using the translating imaging plate detector (TIP) available at the beamline, the TIP set-up provides a furnace operating under vacuum conditions suitable for in-situ XRD in the RT-1000 °C temperature range [50]. The samples were placed in a borosilicate capillary, the XRD patterns were firstly measured at room temperature (static), then during heating at different temperatures: 200 °C, 400 °C, and 600 °C for root samples and 400°, 800°, and 1000 °C for stem ones. During the measurement the capillary rotates to ensure a randomized orientation of the crystallites in the sample. X-ray beam wavelength was λ = 0.82594 Å, XRD patterns were collected in the 2–120° 2θ range. The XRD data were integrated by the Fit2D software [51] and converted to the .xrdml format using the PowDLL software [52]. All XRD patterns were analyzed by the X’Pert HighScore Plus software in order to identify the crystallographic phase composition.

## 4. Results

### 4.1. Chemical Characterization

Selected major and minor element concentrations are reported in Table 1. Rhizosphere samples from Rio Irvi and Rio Naracauli show similar concentrations of Mg (3360 and 2680 ppm, respectively), Na (2510 and 2390 ppm, respectively), and K (12,600 and 16,500 ppm, respectively) while they significantly differ in metals linked to the ore mineralization. Rio Irvi rhizosphere shows a higher concentration of Fe (71000 ppm) Mn (5900), and Zn (26,000 ppm) than Rio Naracauli ones (Fe = 53,800 ppm, Mn = 1800 ppm, and Zn = 18,300 ppm). Conversely, the Pb concentration is extremely higher in Rio Naracauli (53,600 ppm) than in Rio Irvi (1900 ppm). In the plant tissues, the higher concentrations of Fe, Mn, Zn, and Pb are found in the external root and tend to decrease moving to the internal root and to the stem. An exception is represented by Fe and Mn whose concentrations increase from internal root to stem in *Juncus acutus* from the Rio Irvi. The relative concentration of metals in the different tissues of the *Juncus acutus* indicates that the translocation of metals within the plant is not very efficient (see Medas et al. [20] for more details).

### 4.2. Thermal Analysis

**Figure 3:**
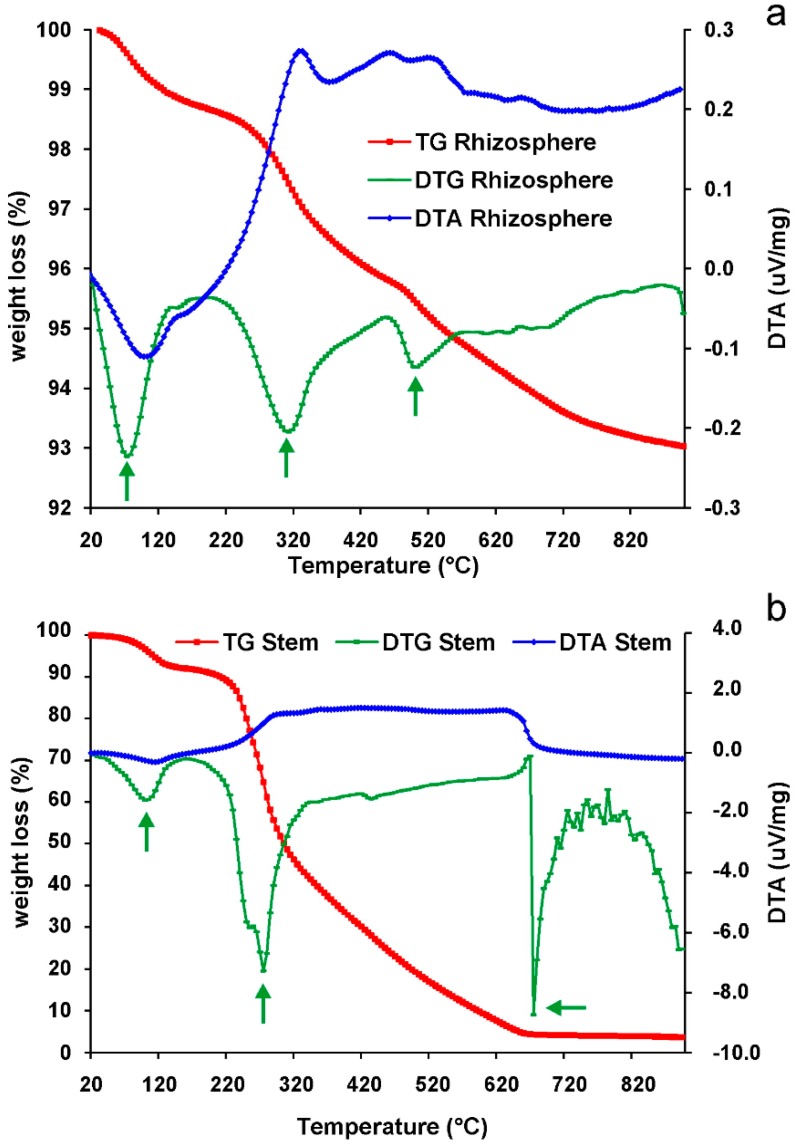
Results of thermal analyses on the rhizosphere (**a**) and the stem (**b**) from Rio Irvi samples. Green arrows indicate the main negative peaks in the derivative of the thermogravimetry (DTG) curves.

The diagrams reporting TG, DTG, and DTA for rhizosphere and stem samples are shown in Figure 3a,b. Samples highlight a different behavior between themselves: the rhizosphere undergoes a total weight loss lower than 10% at the end of the experiment whereas the stem is completely burned showing a total weight loss of ≈ 96 wt. %. The thermal-analysis curves show a small weight loss (about 1 and 3 wt. % for the rhizosphere and the stem, respectively) at low temperatures in association with an endothermic reaction (see DTA curve in Figure 3a,b), which may be attributed to surface-water effects. Therefore, except for this occurrence, two significant events take place in both the rhizosphere and stem samples, as highlighted by the DTG curves. At about 120°C, a decrease of weight loss rate occurs and remains quite constant up to 220 °C. Then, a new increase of weight loss rate, having its maximum velocity around 300 °C, can be observed. This increase is associated with an exothermic reaction as shown by DTA curves. The last significant variation in weight loss was achieved between 400 and 500 °C (stem and rhizosphere, respectively) until the end of the measurement. In this temperature range, the DTG peaks have indicated that the main weight loss, was reached more slowly in the stem sample. The last significant variation of the weight loss rate is observed at about 500 °C in the rhizosphere samples and at 680 °C in the stems. These significant events accompanied by the occurrence of large and asymmetric peaks, highlighted by the DTG curves, are interpreted as a result of phyllosilicate dehydroxylation. Higher temperatures do not produce significant effects in the stems because the combustion of the stem samples is completed (weight loss ≈ 95 wt. % of the initial weight). Rhizosphere samples undergo a quite constant weight loss up to 720 °C, and then this process becomes slower at even higher temperatures. The weight loss steps, and the related endothermic and exothermic reactions as shown by DTA curves. Exothermic reactions occurring in both stem and rhizosphere samples above 220° can be associated to the combustion for the former and to a mineralogical reaction for the latter.

Following the results of thermal analysis, three different temperatures of 120°, 300°, and 600 °C, approximately corresponding to the changes in weight loss rate, were chosen for the ex-situ and in-situ heating of samples to analyze by XRD.

### 4.3. XRD Characterization of the Rio Irvi Samples

Samples of rhizosphere, external surface of roots (epidermis), internal roots, and stems were analyzed by XRD before and after ex-situ thermal treatment. Detected inorganic and organic phases are summarized in Table 2.

**Figure 4:**
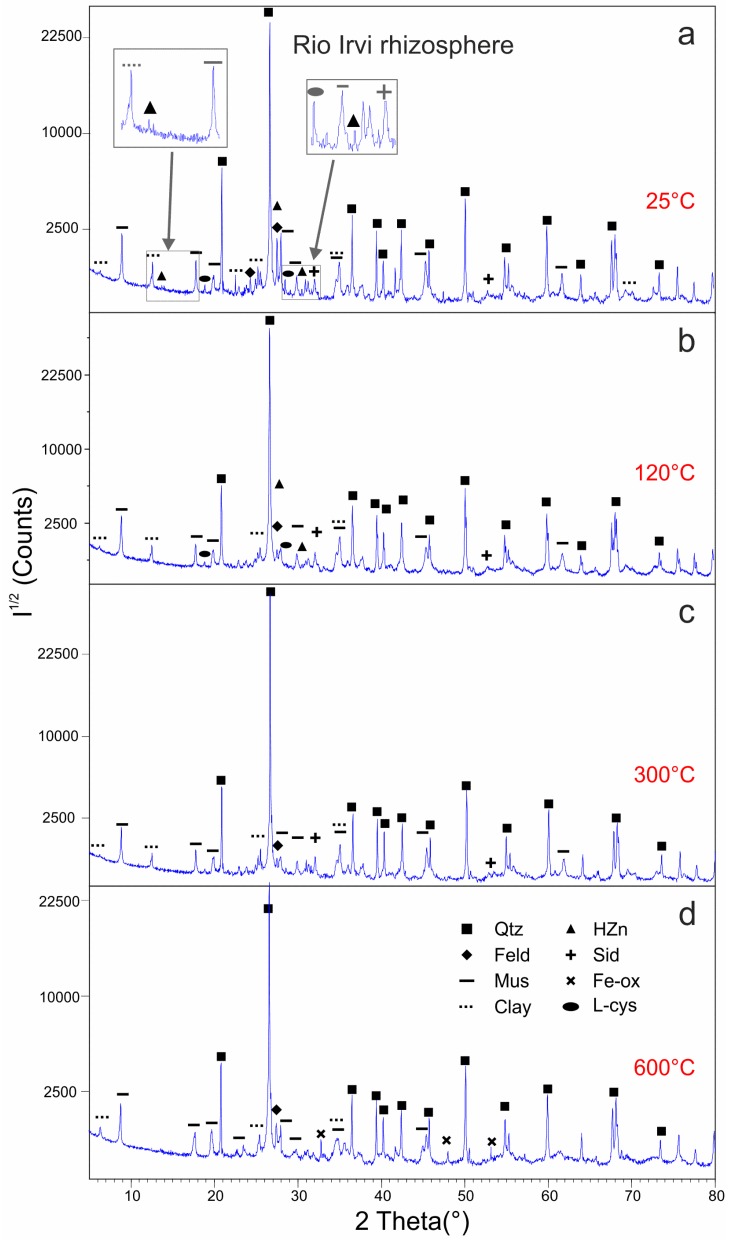
Comparison of X-ray Diffraction (XRD) patterns collected on rhizosphere from Rio Irvi before (**a**) and after ex-situ heating (**b**–**d**). Grey boxes in (**a**) show a close-up of the main hydrozincite peaks. Intensities (I^1/2^) are reported in square root scale. Abbreviations as in Table 2 and Table 3.

The rhizosphere unheated sample contains quartz, muscovite, and microcline which are considered as the main constituents of the bedrock, along with kaolinite, siderite, clinochlore, L-cysteine, and hydrozincite (Figure 4a) whose significance will be discussed in the Discussion. A better mineralogical characterization of phyllosilicates was obtained by XRD with longer acquisition times for the different granulometric fractions of rhizosphere, discriminated by wet gravity separation. These analyses confirm the presence of kaolinite in the clayey fraction and reveal the presence of clinochlore in the coarser one.

Heating at 120 °C slightly modifies the intensity of several peaks of different phases, especially those of hydrozincite, producing the disappearance of the peak at 13.3° 2θ and the decrease of the peak at 30.4° 2θ (see zoom boxes in Figure 4a,b). At 300 °C (Figure 4c), the L-cysteine and hydrozincite are completely degraded (no XRD features detected) while the other phases are still stable. At 600 °C (Figure 4d), the dehydroxylation of kaolinite occurs with the formation of metakaolin and at the same time the intensity of the 001 reflection of chlorite increases (≈6.38° 2θ). Microcline is replaced by its polymorph orthoclase, which is more stable at 600°C and the iron oxide (Fe_2_O_3_) forms at the expense of siderite. Zincite (ZnO), which commonly forms as a product of the thermal decomposition of hydrozincite, is not observed.

**Figure 5:**
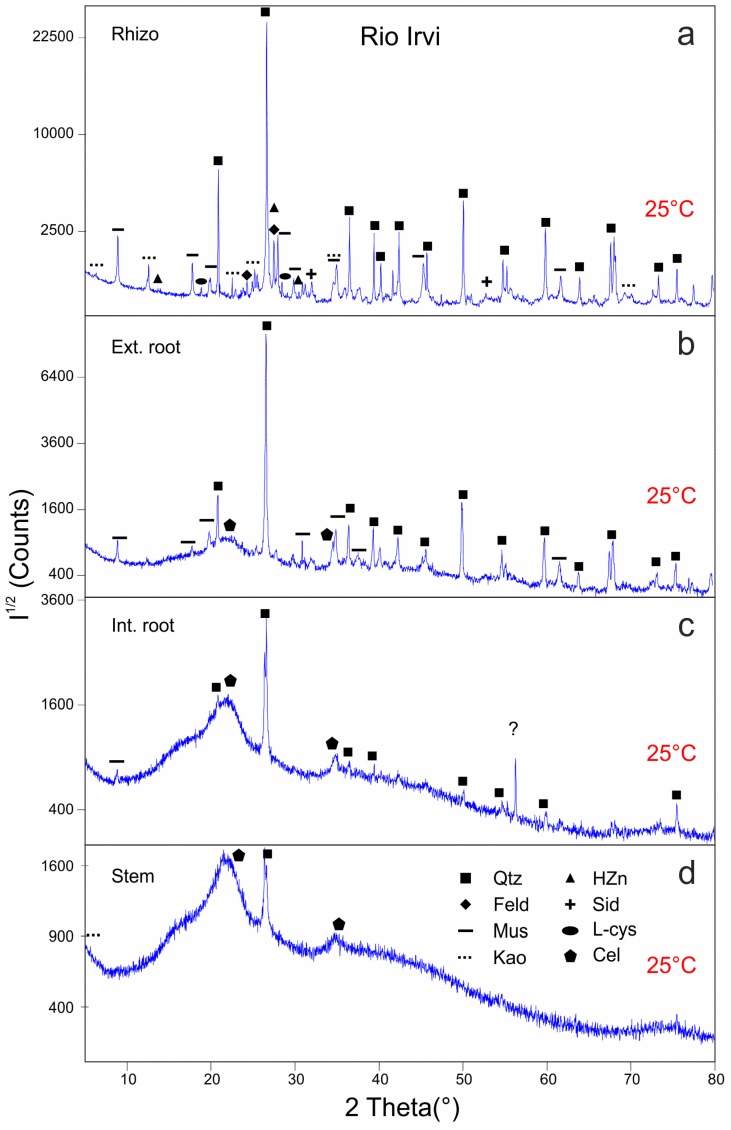
XRD patterns of samples from Rio Irvi collected before heating (25 °C): rhizosphere (**a**), external root (**b**), internal root (**c**), and stem (**d**). Intensities (I^1/2^) are reported in a square root scale to highlight minor peaks. Abbreviations as in Table 2 and Table 3. Question mark in (**c**) represents an unidentified peak probably due to an organic compound (see below).

The external roots (Figure 5b), despite being previously washed with de-ionized water, show the peaks of quartz and muscovite, probably due to their strong adhesion to the roots. Furthermore, a poorly-defined, large peak ranging between 21–25° 2θ testifies the presence of a poorly crystalline phase, likely an amorphous cellulose [53]. During the heating ramp (Table 2), cellulose signal tends to decrease. The first effect of heating (at 120 °C, Figure S1b) is the formation of some new peak that can be assigned to a muscovite polytype that disappears at 300 °C (Figure S1c). XRD pattern after heating at 600 °C (Figure S1d) shows the lack of cellulose peaks due to its combustion and the formation of new phases such as calcium oxide (CaO), carbon (C), sylvite (KCl), anhydrite (CaSO_4_), and albite (Table 2).

XRD patterns of internal roots and stems (Figure 4c,d), as compared to rhizosphere and external root samples, are characterized by large, poorly-defined and less intense peaks due to the increase of cellulose content and to the reduced crystallinity of the mineral phases. In the internal root, as well as in the stems, quartz, cellulose, and muscovite are found. Other phases are not detected but could be masked by the relatively high noise and background levels. In the internal roots, an unassigned peak is found at about 56° 2θ (Figure 5c). It probably belongs to a hydrated phase or to an organic compound given that it disappears at 120 °C (Figure S2b). At 600 °C, cellulose is totally destroyed, and several symmetric small peaks are detected in both stem and internal root samples (Figures S2d and S3d). The former is characterized by very intense sylvite peaks and subordinate quartz, MgO, arcanite/aphthitalite ((K_2_SO_4_/(K,Na)_3_Na(SO_4_)_2_), and willemite (Zn_2_SiO_4_) (Table 2). The latter consists of quartz, arcanite, sylvite, willemite, and muscovite dehydroxylated having the strongest peak at 27.99° 2θ [54].

### 4.4. XRD Characterization of Rio Naracauli Samples

Samples from the Rio Naracauli site were analyzed at room temperature and during in-situ heating at the MCX line of the Elettra synchrotron (Trieste, Italy). Mineral and organic compounds are summarized in Table 3. When performing these analyses, the thermogravimetric data were not yet acquired, thus the steps for the heating ramp were arbitrarily chosen as 200 °C, 400 °C, and 600 °C for root samples (Figure S4a–d) and 400°, 800°, and 1000°C for stem ones (Figure S4a–d). In order to compare Rio Irvi and Rio Naracauli XRD patterns, we emphasize that the X-ray wavelength used for synchrotron radiation XRD analysis was λ = 0.82594 Å giving rise to a compression of about a factor 2 of the XRD patterns with respect to those measured in laboratory with the Cu Kα radiation (λ = 1.54056 Å).

**Figure 6:**
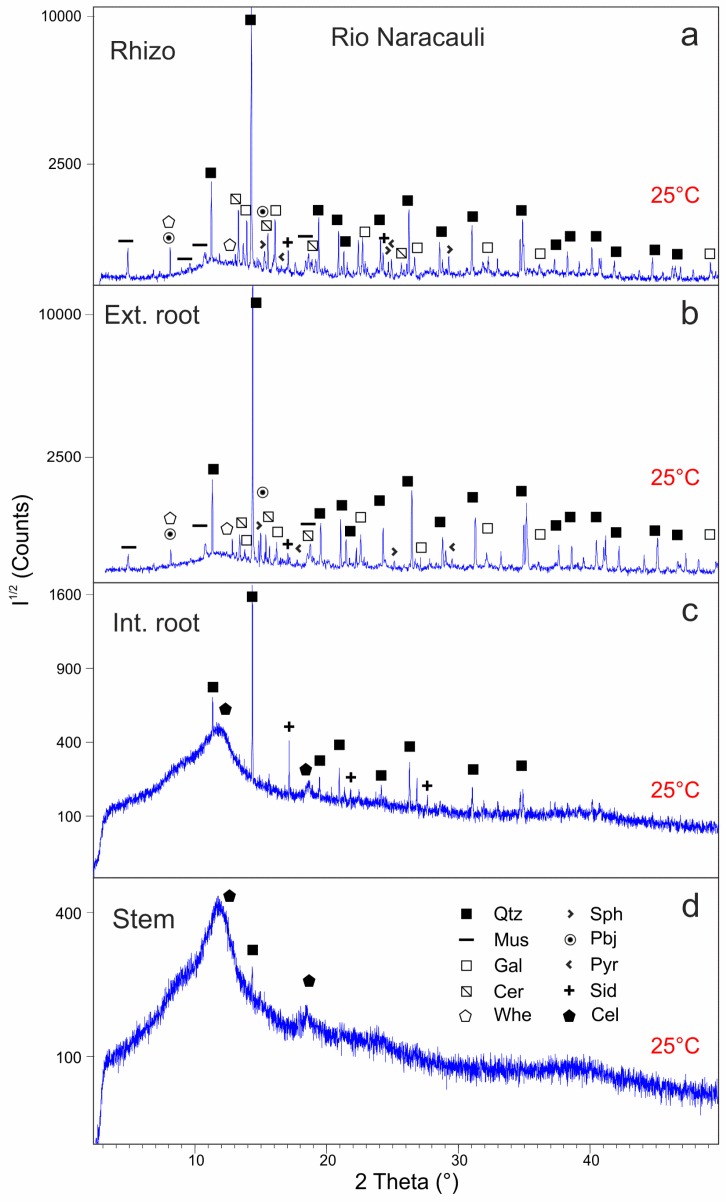
XRD patterns of samples from Rio Naracauli collected before heating (25 °C): rhizosphere (**a**), external root (**b**), internal root (**c**), and stem (**d**). Intensities (I^1/2^) are reported in a square root scale. Abbreviations as in Table 2 and Table 3.

The rhizosphere sample analyzed at room temperature shows a quite complex mineralogy formed by quartz, muscovite, and several phases linked to the mineralization as galena, sphalerite, cerussite, plumbojarosite, siderite, and pyrite (Figure 6a). Whewellite is likely present, as reported by Medas et al. [20], but its main peaks are juxtaposed to those of plumbojarosite leading to some uncertainty in peak attribution. Heating of this sample was not required because the organic matter was totally absent.

The mineral assemblage of the external roots is quite similar to that of the rhizosphere, but the main peaks of the minor phases are commonly less intense and less sharp (Figure 6b). The presence of cellulose or other similar organic compounds is highlighted by the increase of the background level and by a large hump between 11° and 13° 2θ.

During the in-situ heating, quartz peaks become progressively more defined and tend to shift toward lower angles due to its thermal expansion. At 600 °C (Figure S4d), the maximum shift of quartz peaks is reached (0.13° 2θ for the (100) reflection and 0.17° 2θ for the (101) one) and also their relative intensity is significantly modified with a marked increase of the reflections (100) at 11.1° and (112) at 26.2° 2θ and a slight decrease of the most intense one (101) at 14.2° 2θ. However, the transition from α- to β-quartz, commonly occurring around 573°C, does not seem to be accomplished since it involves even higher deformations of lattice parameters that result in stronger shift of the peaks than those here observed [55]. Muscovite dehydroxylation occurs at 600 °C and the intensity of the diffraction peaks are strongly modified. Pyrite and sphalerite crystallinity is enhanced with rising temperatures up to 400 °C (Figure S4c), as seen by their main reflections at 17.5° 2θ for pyrite and 15.2° and 25.0° 2θ for sphalerite. Further temperature increase at 600 °C produces the opposite effect, reducing the peak intensity to prior values. Plumbojarosite and whewellite are found up to 200 °C, while being totally lacking at 400 °C, thus their stability is comprised in this temperature range. At the last temperature step (600 °C), two significant peaks, not detected before, are found at 7.5° and 14.6° 2θ. They roughly correspond to albite major peaks, although shifted due to the thermal expansion. It is supported by the fact that albite has been found, at the same temperature, also in the external and internal roots of Rio Irvi.

In the internal roots (Figure 6c and Figure S5), the presence of cellulose is highlighted by the large “hump” between 10° and 13° 2θ and by the smaller, poor-defined peak at 17.5–18.5° 2θ. Mineral phases are mainly quartz and siderite whose characteristic peak at 17° 2θ is well defined. Residue peaks can be tentatively assigned to feldspars or hydrated sulfate like alunogen, but the high background produced by cellulose likely hide minor peaks of these phases impeding a precise attribution. Furthermore, is not unlikely the presence of clayey minerals or of whewellite [20], but their presence cannot be detected. The progressive heating of internal root samples does not reduce significantly the noise produced by cellulose, even at the maximum temperature. As observed in the external root samples, the heating produces a shift and the change of relative intensities of quartz reflections. In addition, the disappearance of siderite above 400 °C, and the crystallization of small amounts of CaO are observed.

In the stem sample, cellulose and quartz can be detected at room temperature (Figure 6d). To reduce the noise of cellulose, a different heating ramp for the stem samples was chosen respect to the roots, increasing temperature at 400°, 800°, and 1000° (Figure S6). Nonetheless, this procedure failed in improving the signal, involving just an increase and a more marked shift of quartz peaks.

## 5. Discussion

The rhizosphere is the part of the soil ecosystem where plant roots, soil, and microorganisms interact with each other [56,57]. Here, the metabolic processes of roots create a peculiar physicochemical environment that often differs from that of the bulk soil [58,59]. Rhizosphere is characterized by the presence of several mineral and organic phases. In our study, quartz, phyllosilicates, feldspars, and ore-metal sulfides are probably inherited from the bedrock, whereas other phases are the product of biological and/or geochemical secondary processes. Heavy metal sulfates and carbonates result from the alteration and oxidation of the Pb–Zn–Fe mineralization as well as kaolinite results from feldspar weathering. Other minerals could derive from the biological activity within the rhizosphere i.e., hydrozincite, whose crystallization can be promoted by the activity of bacteria (*Scytonema* sp.) and microalgae (*Chlorella* sp.) [60,61]. Biologically-induced precipitation results in a poorly crystalline hydrozincite characterized by weak, broad, and shifted peaks [62,63] as those observed in this study and detected with long acquisition times (about 8 hours). However, XRD data are not enough to state with certainty the biological origin of this mineral that could also precipitate by geochemical processes.

The presence of hydrozincite in rhizosphere samples of *Juncus acutus* from Rio Irvi is in agreement with the X-ray Absorption Near Edge Structure (XANES) data of Medas et al. [20]. According to these authors, about 50% of Zn is bound to hydrozincite, whereas the remaining part is distributed in Zn sulfate hydrate and in metal-organic compounds. This is probably the reason why such a significant amount of Zn (26,000 mg/kg) is barely detected by XRD analyses. As reported in several occurrences [64], siderite also could be a product of bio-precipitation but, in this case, it is more likely a mineral belonging to the bedrock given that it is quite common in the gangue of the study area. The biological and chemical activity of rhizosphere is further testified by the presence of L-cysteine root exudate [65,66], an amino acid synthesized by plants for detoxification [67] and immunity by pathogens [68,69].

The first effect of rhizosphere heating is the decomposition of L-cysteine and hydrozincite before 300 °C. Total decomposition of L-cysteine at 300 °C is in agreement with previous studies [70,71] and it is preceded by the release of volatile compounds starting from 200 °C [72]. Hydrozincite as well, is reported to decompose in the 220–250 °C temperature range [73,74], resulting in zincite crystallization. In this case, neither zincite nor other Zn phases are observed after the hydrozincite decomposition, even at 600 °C. Siderite decomposition commonly starts above 450 °C [75] but the particle size or the chemical composition can significantly modify this temperature [76]. In this case study, siderite is still stable at 300°C and becomes unstable at 600 °C, temperature at which iron oxide was found. This latter is probably attributable to a siderite thermal decomposition product and as a result of dehydroxylation of amorphous goethite, commonly found in the soil of studied area. Other significant changes occur over 600 °C: the disappearance of the strongest kaolinite peak at about 12.5° 2θ, due to its dehydroxylation [77,78]; the intensity increase of a peak at 6.4° 2θ, that could be related to clinochlore crystallization; and finally, the slight shift of K-feldspar peaks which suggest the microcline-orthoclase transformation.

The external roots from the two sites show a mineral assemblage similar to that of the corresponding rhizosphere. Maybe, these minerals were incorporated into the organic tissues during the plant growth as observed by [30]. In both XRD patterns, the cellulose signal is detected. The pattern of cellulose here reported seems to suggest the presence of both microcrystalline and amorphous cellulose or of a polymer with intermediate crystallographic properties. Indeed, all the main peaks of microcrystalline cellulose are found but their base widths and their intensities can be compared with those of the amorphous phase [53]. In particular, the two peaks at 15.02° and 16.49° 2θ are here observed as a poorly defined “humps” comprised in a 2θ range of 14.5–17.5° (with CuKα beam).

**Figure 7:**
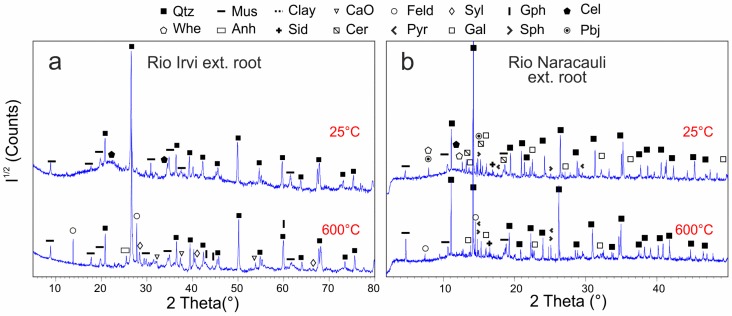
XRD patterns showing the effect of ex-situ heating on external root samples from Rio Irvi (**a**) and in-situ heating on external root samples from Rio Naracauli (**b**). Intensities (I^1/2^) are reported in a square root scale. Abbreviations as in Table 2 and Table 3.

External root samples show a different behavior under ex-situ and in-situ heating (Figure 7a,b). In the first case, no significant changes occur until the last stage (600 °C) at which the organic compounds are totally destroyed and new phases are detected: carbon as product of cellulose combustion, CaO and anhydrite resulting from calcination and dehydration of the Ca-bearing phases, sylvite and plagioclase. On the contrary, in-situ heating produces only minor changes suggesting that: (i) the capillary sample holder does not allow the air flux and thus the combustion and the dehydration of the sample; (ii) the cooling down procedure in ex-situ experiments, not occurring in in-situ ones, could have a role that should be considered. The main effect revealed by in-situ measurements is the plumbojarosite and whewellite disappearance. The latter is one of the most common phases in plants tissues, commonly occurring within the cells but also in extracellular form [79,80]. Whewellite is stable up to 400 °C as already reported by Quintana et al. [81] and above this temperature it decomposes, probably forming calcite [82,83]. Further temperature increases result in the appearance of two small unassigned peaks, but expected reactions as cellulose combustion or sulfides oxidation do not occur.

**Figure 8:**
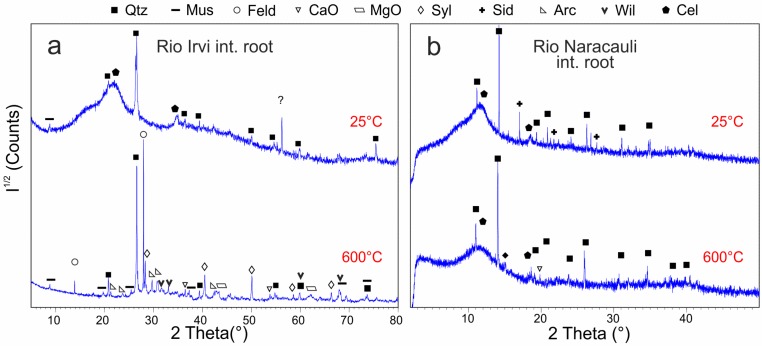
XRD patterns showing the effect of ex-situ heating on internal root samples from Rio Irvi (**a**) and in-situ heating on internal root samples from Rio Naracauli (**b**). Intensities (I^1/2^) are reported in a square root scale. Abbreviations as in Table 2 and Table 3.

In the internal root and in the stem the main compound is the cellulose but also quartz and muscovite are commonly detected. At room temperature, no crystalline metal-bearing phases were found, probably because the metals are bound to organic ligands [35,36] or are in amorphous phases. Plant tissues react in different ways to the ex-situ or in-situ heating (Figure 8a,b). The latter does not produce significant changes even at temperature as high as 1000 °C at which cellulose background is slightly reduced but not enough to observe the reflections relative to minor minerals. The former produces the first changes since the first steps of heating ramp as the disappearance of a peak at 56° 2θ (probably related to a hydrated phase) and the formation of sylvite and graphite at 300 °C. After heating at 600 °C, cellulose is totally destroyed and new K-bearing phases, i.e., arcanite (K_2_SO_4_) and sylvite (KCl) crystallize probably at the expense of the potash hosted in organic tissues. Feldspars also are found and are revealed by different peaks in the 27–28° 2θ range. Plagioclase, together with sylvite and arcanite has been reported from pyrolysis of organic wastes aimed to convert them in biochar [84,85]. Furthermore, at 600 °C, willemite (Zn_2_SiO_4_) is observed in roots and stems whereas calcium and magnesium oxides are detected only in the stem. Although no Zn phases were detected before heating, and it is known that *Juncus acutus* is not a Zn accumulator plant because it tends to concentrate Zn in the root epidermis [20], the presence of willemite is not unlikely. Chemical analysis show Zn concentrations of 9700 mg/kg in the internal root and 1800 mg/kg in the stem so, considering that after heating at 600 °C about 95% of the initial mass is loss, it is not surprising to detect traces of Zn phases in the combustion products. The appearance of willemite after heating is in agreement with the stability of this phase at temperatures higher than 250 °C (at room pressure), whereas at lower temperatures hydrated Zn silicates such as sauconite (Zn_3_Si_4_O_10_(OH)_2_·nH_2_O) or hemimorphite (Zn_4_(OH)_2_Si_2_O_7_·H_2_O) are more stable [86,87].

As mentioned above, the thermal treatment of biomasses is widely studied to evaluate their potential in phytomining [37,38], and to optimize the production of biochar [32,39] and biofuel [88,89]. Ecological outcomes vary from the amendment/bioremediation effect onto the soil [33,90,91,92], to the CO_2_ sequestration [93,94], to the energy saving resulting from the use of these renewable sources [84], and to the metal recovery [37].

The chemical, physical, and mineralogical features of the pyrolyzed or combusted biomasses, and thus their efficiency, are mainly controlled by the treatment conditions (temperature, heating rate, duration, and gas/water presence) and by the starting material [39,95]. For this reason, several papers report detailed characterization of biomasses before and after thermal treatment, but few of them highlight the great relevance of the mineralogical composition. As reported by Vassilev et al. [96] (and references therein), great interest is focused on the chemical composition of biomasses but less attention is paid to the specific phases and minerals. The knowledge of the latter is essential to assess the bioavailability of nutrients [97] and the metal removal capacity of biochar [98], as well as for the metal recovery from biomass ashes [99] (and references therein). Furthermore, the influence of mineral phases content in biomass ashes has been related to the efficiency and to the various technical issues occurring in power plants that use this kind of fuel [100].

## 6. Conclusions

In this work, we presented not only a specific case study of plant/soil interaction in an abandoned mine area contaminated by metals, but also a methodology that could be applied elsewhere.

The ex-situ heating turned out to be a useful tool in determining the occurrence of metal-bearing phases in plants, reducing or even removing the high background noise level produced by amorphous or low crystalline organic compounds and thus allowing to: (i) recognize minor minerals already present in the samples but previously hidden or barely recognizable and (ii) crystallize new phases reflecting the chemical composition of the samples. This procedure, could be very effective when more sophisticated analytical techniques are not available, especially if combined with thermal analyses in order to reveal the temperature stages at which the significant reactions occur. Indeed, too low temperatures do not allow the development of phase transitions and the decomposition of organic compounds, whereas too high temperatures should lead to the decomposition of the new crystallizing phases before they are detected.

On the contrary, the in-situ high temperature synchrotron-based XRD analyses are more sensitive for revealing very minor phases in mainly-inorganic samples (i.e., soil and rhizosphere), but they are less effective in organic matter-rich ones. Indeed, the in-situ heating failed in improving the quality of signal because, even at extremely high temperature, the organic matter was not destroyed. The absence of oxygen within the capillary sample holder does not allow the cellulose combustion and also impedes the sulfides oxidation. In addition, the shift observed in the XRD patterns, due to the thermal expansion, make it difficult to interpret the peaks. Indeed, each mineral has a different coefficient of thermal expansion and even the same mineral, if not isotropic, is affected by differential expansions.

Further developments of this technique should be aimed at obtaining a more precise attribution of all peaks, for instance by coupling synchrotron XRD analysis of ex-situ heated samples with micro-XRF mapping. A development of this kind of analysis could improve our understanding of the interaction between plants and soil and could also have practical application. The potential recovery of metals by hyper-accumulator plants, that is the object of several studies, could benefit from XRD analysis combined with thermal treatment aimed at discover which temperatures lead to the crystallization of mineral phases more suitable for exploitation. Another potential application of these combined techniques is the production of biochar from vegetal masses. In this case, it is also required to know the optimum temperatures to obtain minerals that easily decompose and make bioavailable their chemical constituents.

## Figures and Tables

**Table 1:** Chemical analysis of selected major and minor elements of collected samples. * indicates data reported from Medas et al. [20].

Sample	Al *	Fe *	Mg	Mn *	Ca	Na	K	Zn *	Pb *
	mg/kg	mg/kg	mg/kg	mg/kg	mg/kg	mg/kg	mg/kg	mg/kg	mg/kg
**Rio Irvi**									
Rhizo	34,200	71,000	3360	5900	5270	2510	12,600	26,000	1900
Ext. root	4200	9100	3480	640	6490	3320	2520	14,900	1400
Int. root	450	740	1850	60	3890	1950	970	9700	850
Stem	600	1400	1790	90	2250	4470	6080	1800	90
**Rio Naracauli**									
Rhizo	40,200	53,800	2680	1800	2290	2390	16,500	18,300	53,600
Ext. root	22,200	30,900	1770	1100	1640	1890	12,100	11,700	24,300
Int. root	2000	2800	220	80	430	510	2560	1600	2100
Stem	120	112	980	14	2020	2170	11,400	290	9.1

**Table 2:** Mineral assemblages of samples from Rio Irvi at the different temperatures of the heating ramp. Abbreviation are: Qtz, quartz; Mus, muscovite; Feld, feldspars; Clay, clayey minerals; Sid, siderite; H-Zn, hydrozincite; Zn-ox, zinc oxides; Fe-ox, iron oxides; Wil, willemite; CaO and MgO, calcium and magnesium oxides; Gph, graphite; Anhy, anhydrite; Arc, arcanite; Syl, sylvite; Cell, cellulose; L-cys, L-cysteine.

Rio Irvi	Qtz	Mus	Feld	Clay	Sid	H-Zn	Zn-ox	Fe-ox	Wil	CaO	MgO	Gph	Anhy	Arc	Syl	Cell	L-cys
**Rhizosphere**																	
25 °C	X	X	X	X	X	X											X
120 °C	X	X	X	X	X												X
300 °C	X	X	X	X	X												
600 °C	X	X	X	X				X									
**Ext. root**																	
25 °C	X	X														X	
120 °C	X	X														X	
300 °C	X	X														X	
600 °C	X	X	X							X		X	X		X		
**Int. root**																	
25 °C	X	X														X	
120 °C	X															X	
300 °C	X	X														X	
600 °C	X	X	X	X					X	X		X		X	X		
**Stem**																	
25 °C	X			X												X	
120 °C	X			X												X	
300 °C	X											X			X	X	
600 °C	X		X						X		X	X		X*	X		

**Table 3:** Mineral assemblages of samples from Rio Naracauli at the different temperatures of the heating ramp. Abbreviation are: Qtz, quartz; Mus, muscovite; Feld, feldspars; Clay, clayey minerals; Gal, galena; Sph, sphalerite; Cer, cerussite; Pbj, plumbojarosite; Sid, siderite; Pyr, pyrite; CaO, calcium oxide; Whe, whewellite; Cell, cellulose.

Rio Naracauli	Qtz	Mus	Feld	Clay	Gal	Sph	Cer	Pbj	Sid	Pyr	CaO	Whe	Cell
**Rhizosphere**													
25 °C	X	X			X	X	X	X	X	X		X	
**External root**													
25 °C	X	X			X	X	X	X	X	X		X	X
200 °C	X	X		X	X	X	X	X	X	X		X	X
400 °C	X	X	X		X	X	X		X	X			X
600 °C	X	X	X	X	X	X	X		X	X			X
**Internal root**													
25 °C	X								X				X
200 °C	X								X				X
400 °C	X								X		X		X
600 °C	X										X		X
**Stem**													
25 °C	X												X
400 °C	X												X
800 °C	X												X
1000 °C	X												X

## Supplementary Materials

The following are available online at https://www.mdpi.com/1660-4601/16/11/1976/s1.
