# Supplementary material for: XRD-Thermal Combined Analyses: An Approach to Evaluate the Potential of Phytoremediation, Phytomining, and Biochar Production
Source: Int J Environ Res Public Health. 2019 Jun 4;16(11):1976. doi: 10.3390/ijerph16111976 (PMC6603955; doi:10.3390/ijerph16111976)
Supplement: Supplementary file 1 [file ijerph-16-01976-s001.pdf]

International Journal of
Environmental Research and Public Health

MDPI

Supplementary Material

XRD-Thermal Combined Analyses: an Approach to Evaluate the Potential of Phytoremediation, Phytomining and Biochar Production

Dario Fancello [1], Jessica Scalco [1], Daniela Medas [1,*], Elisa Rodeghero [2], Annalisa Martucci [2], Carlo Meneghini [3] and Giovanni De Giudici [1]

[1] Department of Chemical and Geological Sciences, University of Cagliari, Cittadella Universitaria, Monserrato, 09042, CA – Italy;
[2] Department of Physics and Earth Sciences, University of Ferrara, Ferrara, I-44100, Italy;
[3] Department of Sciences, University Roma Tre, 00146, Rome, Italy;

* Correspondence: dmedas@unica.it

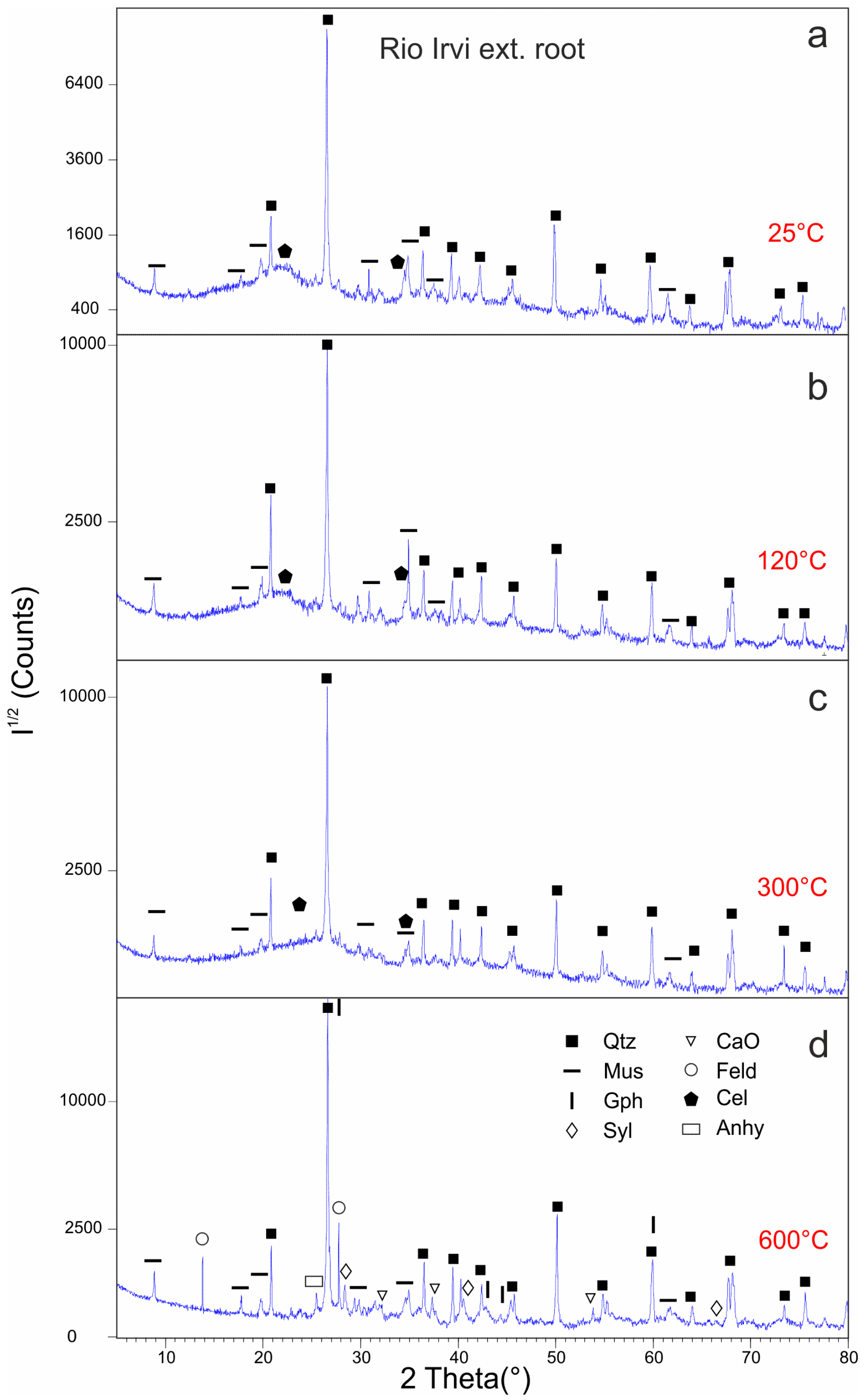

Figure S1. Comparison of XRD patterns collected on external roots from Rio Irvi before (a) and after *ex-situ* heating (b-d). Intensities ($I^{1/2}$) are reported in square root scale. Abbreviation are: Qtz, quartz; Mus, muscovite; Gph, graphite; Syl, sylvite; CaO, calcium oxide; Feld, feldspars, Cell, cellulose; Anhy, anhydrite.

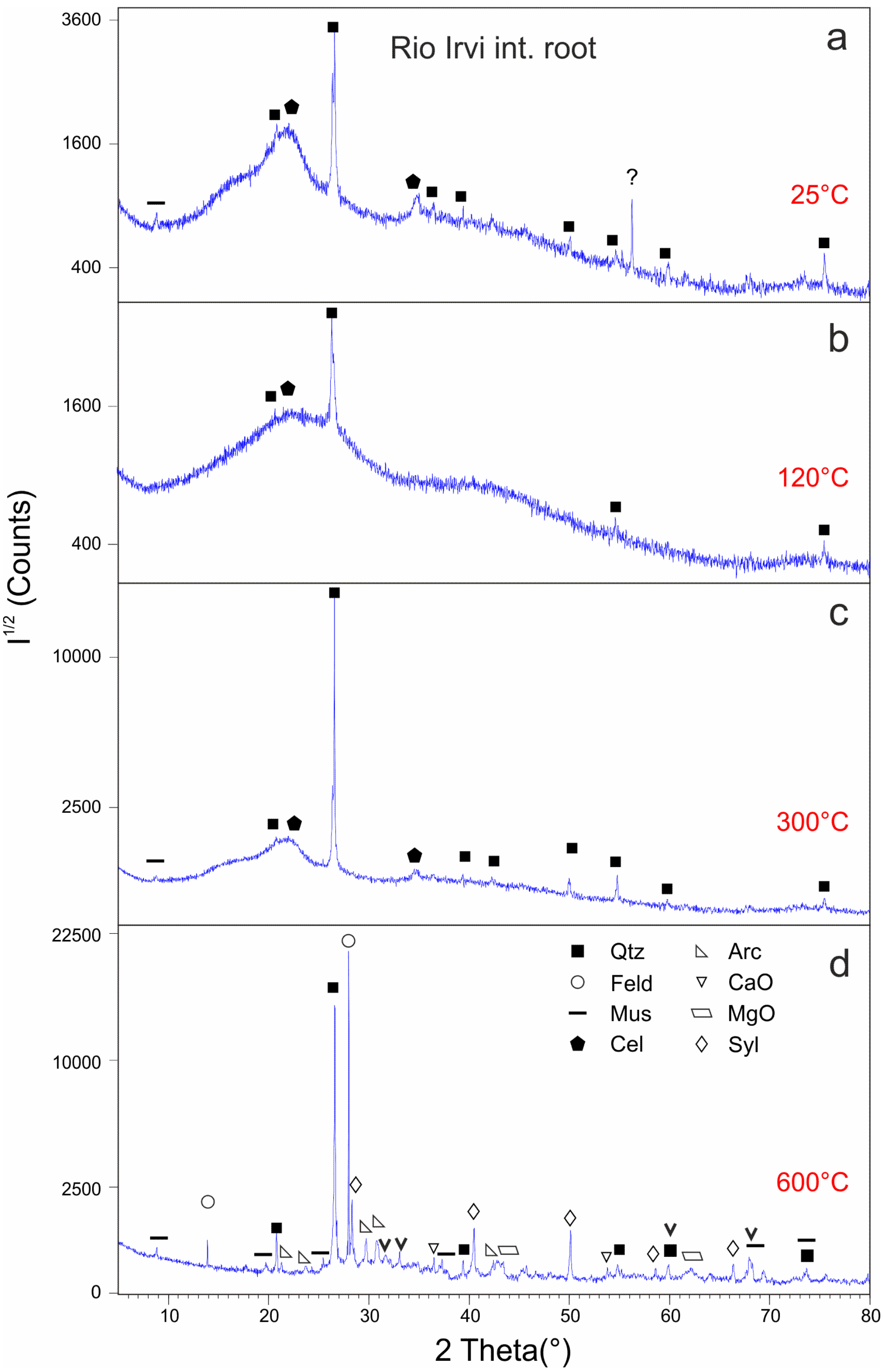

Figure S2. Comparison of XRD patterns collected on internal roots from Rio Irvi before (a) and after *ex-situ* heating (b-d). Intensities ($I^{1/2}$) are reported in square root scale. Abbreviation are: Qtz, quartz; Feld, feldspars, Mus, muscovite; Cell, cellulose; Arc, arcanite; CaO, calcium oxide; MgO, magnesium oxide; Syl, sylvite.

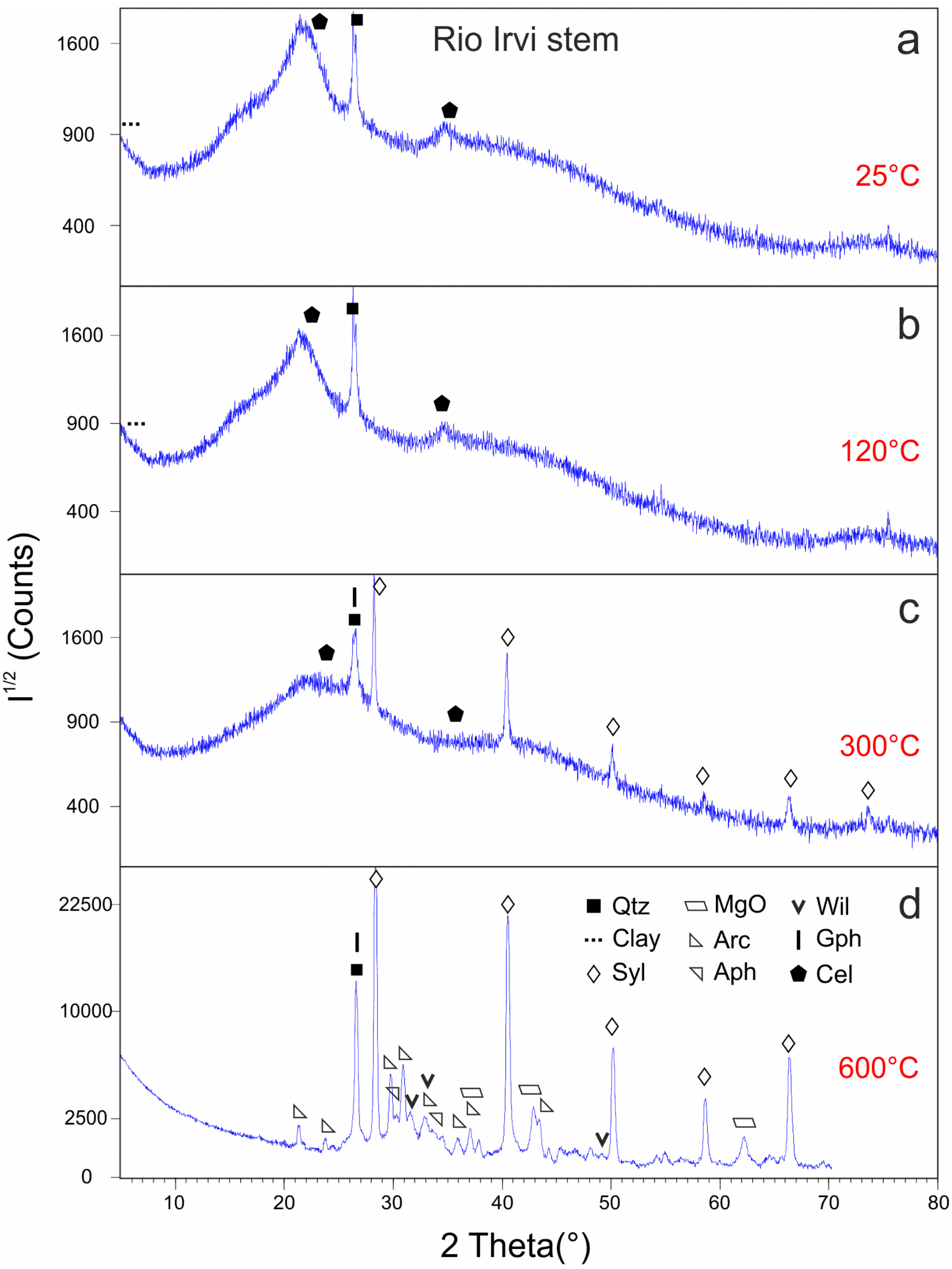

Figure S3. Comparison of XRD patterns collected on stems from Rio Irvi before (a) and after *ex-situ* heating (b-d). Intensities ($I^{1/2}$) are reported in square root scale. Abbreviation are: Qtz, quartz; Clay, clayey minerals; Syl, sylvite; MgO, magnesium oxide; Arc, arcanite; Aph, aphthitalite; Wil, willemite; Gph, graphite; Cell, cellulose.

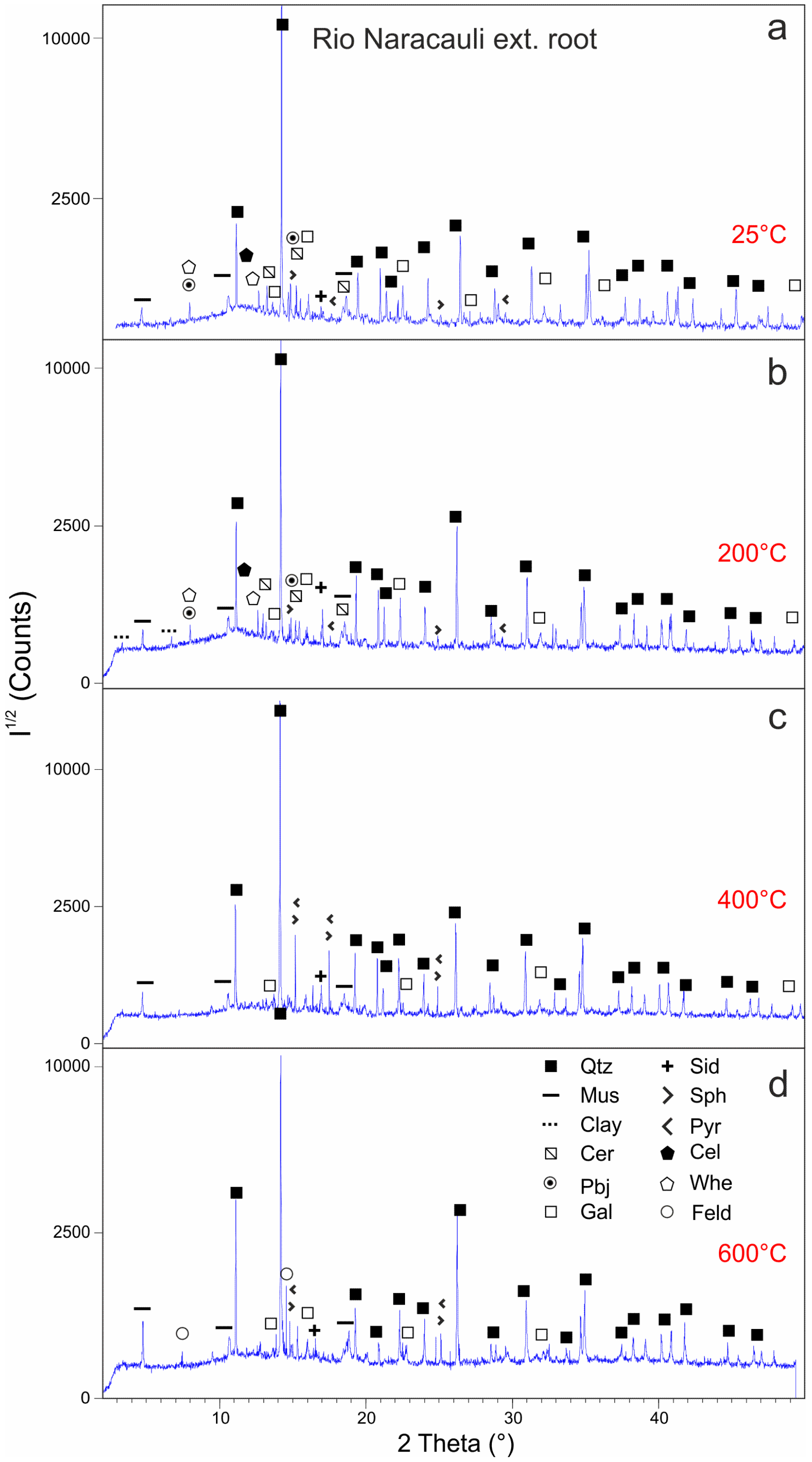

Figure S4. Comparison of XRD patterns collected on external roots from Rio Naracauli before (a) and after *in-situ* heating (b-d). Intensities ($I^{1/2}$) are reported in square root scale. Abbreviation are: Qtz, quartz; Mus, muscovite; Clay, clayey minerals; Cer, cerussite; Pbj, plumbojarosite; Gal, galena, Sid, siderite; Sph, sphalerite; Pyr, pyrite; Cel, cellulose; Whe, whewellite; Feld, feldspars.

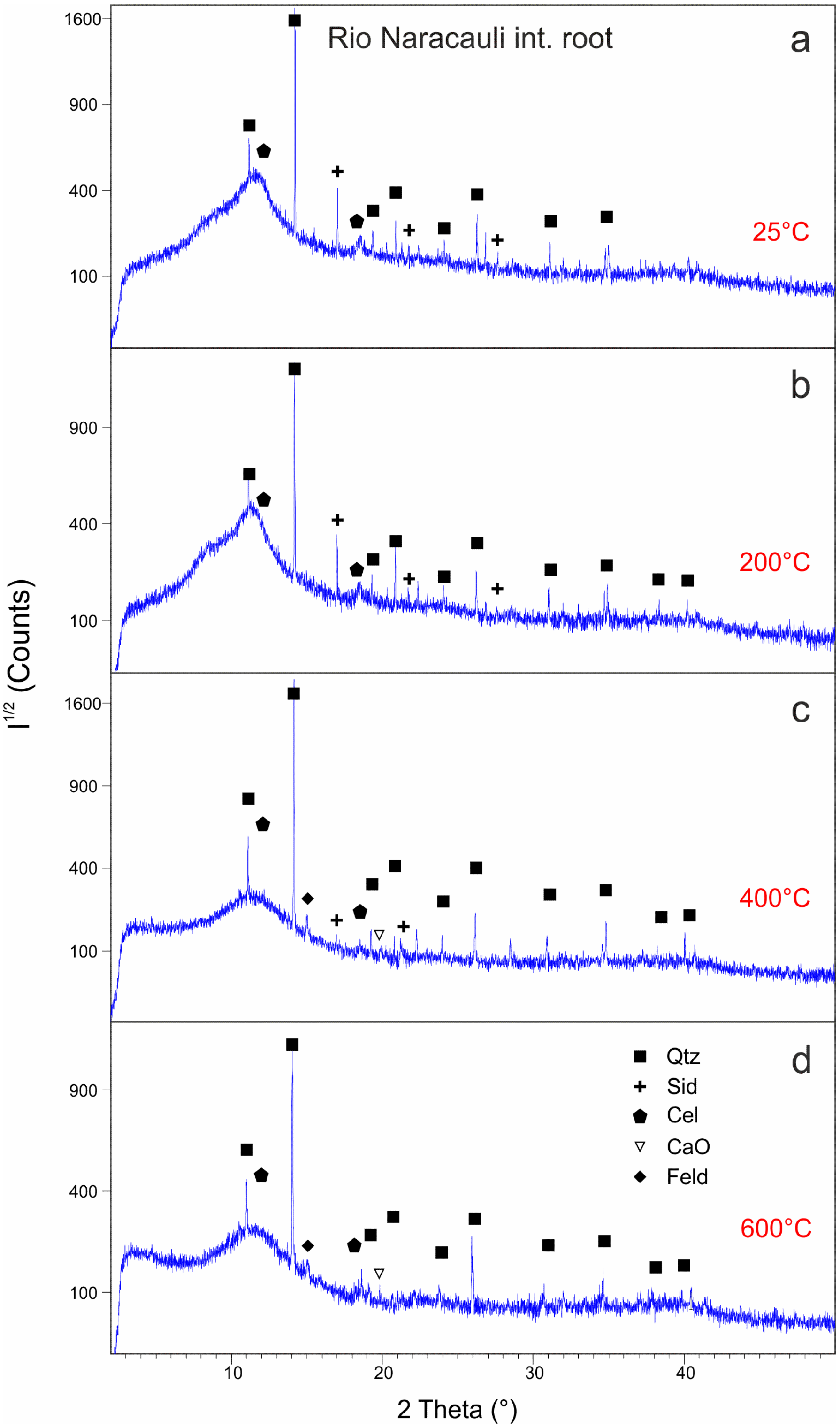

Figure S5. Comparison of XRD patterns collected on internal roots from Rio Naracauli before (a) and after *in-situ* heating (b-d). Intensities ($I^{1/2}$) are reported in square root scale. Abbreviation are: Qtz, quartz; Sid, siderite; Cel, cellulose; CaO, calcium oxide; Feld, feldspars.

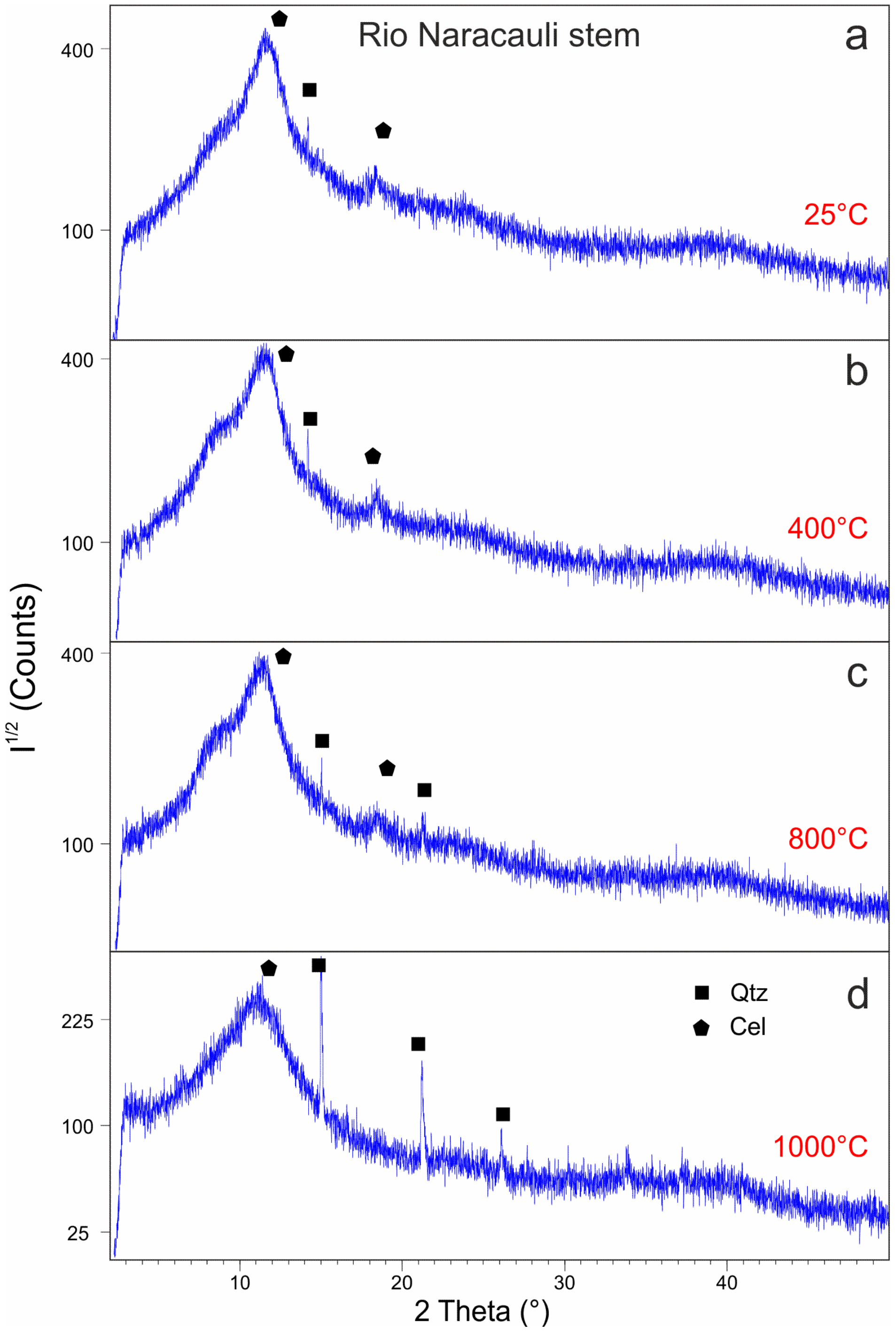

Figure S6. Comparison of XRD patterns collected on stems from Rio Naracauli before (a) and after *in-situ* heating (b-d). Intensities ($I^{1/2}$) are reported in square root scale. Abbreviation are: Qtz, quartz; Cel, cellulose.
